# Classification of Lung Disease in Children by Using Lung Ultrasound Images and Deep Convolutional Neural Network

**DOI:** 10.3389/fphys.2021.693448

**Published:** 2021-08-27

**Authors:** Silvia Magrelli, Piero Valentini, Cristina De Rose, Rosa Morello, Danilo Buonsenso

**Affiliations:** ^1^Independent Researcher, Zurich, Switzerland; ^2^Department of Woman and Child Health and Public Health, Fondazione Policlinico Universitario A. Gemelli IRCCS, Rome, Italy; ^3^Global Health Research Institute, Istituto di Igiene, Università Cattolica del Sacro Cuore, Rome, Italy; ^4^Dipartimento di Scienze Biotecnologiche di Base, Cliniche Intensivologiche e Perioperatorie, Università Cattolica del Sacro Cuore, Rome, Italy

**Keywords:** deep-learning CNN, bronchiolitis, pneumonia, children, lung ultrasonography

## Abstract

Bronchiolitis is the most common cause of hospitalization of children in the first year of life and pneumonia is the leading cause of infant mortality worldwide. Lung ultrasound technology (LUS) is a novel imaging diagnostic tool for the early detection of respiratory distress and offers several advantages due to its low-cost, relative safety, portability, and easy repeatability. More precise and efficient diagnostic and therapeutic strategies are needed. Deep-learning-based computer-aided diagnosis (CADx) systems, using chest X-ray images, have recently demonstrated their potential as a screening tool for pulmonary disease (such as COVID-19 pneumonia). We present the first computer-aided diagnostic scheme for LUS images of pulmonary diseases in children. In this study, we trained from scratch four state-of-the-art deep-learning models (VGG19, Xception, Inception-v3 and Inception-ResNet-v2) for detecting children with bronchiolitis and pneumonia. In our experiments we used a data set consisting of 5,907 images from 33 healthy infants, 3,286 images from 22 infants with bronchiolitis, and 4,769 images from 7 children suffering from bacterial pneumonia. Using four-fold cross-validation, we implemented one binary classification (healthy vs. bronchiolitis) and one three-class classification (healthy vs. bronchiolitis vs. bacterial pneumonia) out of three classes. Affine transformations were applied for data augmentation. Hyperparameters were optimized for the learning rate, dropout regularization, batch size, and epoch iteration. The Inception-ResNet-v2 model provides the highest classification performance, when compared with the other models used on test sets: for healthy vs. bronchiolitis, it provides 97.75% accuracy, 97.75% sensitivity, and 97% specificity whereas for healthy vs. bronchiolitis vs. bacterial pneumonia, the Inception-v3 model provides the best results with 91.5% accuracy, 91.5% sensitivity, and 95.86% specificity. We performed a gradient-weighted class activation mapping (Grad-CAM) visualization and the results were qualitatively evaluated by a pediatrician expert in LUS imaging: heatmaps highlight areas containing diagnostic-relevant LUS imaging-artifacts, e.g., A-, B-, pleural-lines, and consolidations. These complex patterns are automatically learnt from the data, thus avoiding hand-crafted features usage. By using LUS imaging, the proposed framework might aid in the development of an accessible and rapid decision support-method for diagnosing pulmonary diseases in children using LUS imaging.

## 1. Introduction

Bronchiolitis is a viral acute lower respiratory-tract infection and the most common reason for hospitalization and intensive-care-unit admission of children worldwide (Choi and Lee, 2012; Øymar et al., 2014).

The diagnosis of infants with bronchiolitis is difficult: there exists no unambiguous definition of the disease; the diagnosis is based on clinical evaluation and anamnesis (Ralston et al., 2014) hence determined by different conditions such as age and variability in the disease state. Furthermore, many of these parameters are based on subjective clinical findings and can be diversely interpreted by different physicians, according to their clinical experience. Finally, bronchiolitis can require serial observations over time, which in turn presents several challenges when performed in emergency departments.

Community acquired pneumonia (CAP) is also pervasive and a frequent cause of pediatric morbidity and mortality (Liu et al., 2015). A diagnosis of CAP, similarly to that of bronchiolitis, relies mainly on medical history and clinical examination. However, these methods suffer from poor sensitivity and specificity hence, to confirm CAP, physicians need to prescribe medical imaging techniques such as chest X-ray (Bradley et al., 2011; World Health Organization, 2014; Shah et al., 2017).

There is growing research interest (Ralston et al., 2014; Collins and Varmus, 2015) in discovering objective parameters that are easy to measure and that could help the physician to perform a more accurate evaluation of children possibly infected with a respiratory disease, thus to make prompt clinical decisions.

Ultrasound technology (US) is one of the most often used imaging diagnostic tools for physicians and radiologists, due to its relative safety, portability, repeatability, cost effectiveness, and operator comfort. Examinations can be carried out, after appropriate training, even by non-specialist radiologists [point-of-care ultrasound, POCUS (Kessler et al., 2017)]. Therefore, US imaging presents several major advantages over other medical imaging modalities such as magnetic resonance imaging (MRI), computed tomography (CT), and X-ray.

In the last few decades, lung ultrasound (LUS) imaging supported clinical examinations for neonatal and pediatric respiratory diseases, as a valid tool for evaluating the lung parenchyma (Dunn and Fry, 1961; Bauld and Schwan, 1974; Volpicelli et al., 2012; Rosenfield et al., 2015). This avoids unnecessary exposure of children to ionizing radiation (Buonsenso et al., 2019a). Several studies have demonstrated the usefulness of LUS imaging in the diagnosis and follow-up of community-acquired pneumonia (Berce et al., 2019; Musolino et al., 2019; Najgrodzka et al., 2019; Buonsenso et al., 2021) and, in particular, of bronchiolitis (Basile et al., 2015; Di Mauro et al., 2019; Supino et al., 2019; Buonsenso et al., 2021).

In an attempt to objectively quantify respiratory distress, many scoring systems were developed on the bases of the visual features generated by the interaction between the ultrasound beam and the lung (Supino et al., 2019; Buonsenso et al., 2020e). The appearance of these features varies according to the specific composition of the lung periphery. In general, the main lung ultrasound features of lung inflammatory diseases include irregular pleural line; short and long vertical artifacts; white-lung; consolidations and effusions. However, despite documented medical evidence (Lichtenstein et al., 1997, 2009; Reißig and Kroegel, 2003; Jambrik et al., 2004; Soldati et al., 2006; Volpicelli et al., 2006; Copetti et al., 2008; Gargani et al., 2008) and extensive acoustic studies (Dunn and Fry, 1961; Bauld and Schwan, 1974; Dunn, 1974, 1986; Pedersen and Ozcan, 1986; Mikhak and Pedersen, 2002; Volpicelli et al., 2012), the interpretation of the lung ultrasound features is subjectively made by the clinician/sonographer. US imaging also presents unique challenges, such as low imaging-quality caused by noise and artifacts, and high inter- and intra-observer variability across different institutes and manufacturers of US systems. To address these challenges, it is essential to develop advanced automatic US image-analysis methods in order to make US diagnosis, assessment, and image-guided interventions/therapy more objective, accurate, and intelligent.

In the past 7 years, deep learning (LeCun et al., 2015), a subfield of machine learning (ML), also due to improvements in device capabilities (computing power, memory capacity, power consumption, image sensor resolution, and optics) has seen a dramatic resurgence, with striking improvements in the performance and cost-effectiveness of vision-based applications (Voulodimos et al., 2018). It solves problems that are beyond human capability or that were previously considered intractable, and it demonstrates huge potential for various automatic tasks in medical-image analysis (Greenspan et al., 2016; Litjens et al., 2017; Shen et al., 2017; Ker et al., 2018). Therefore, it receives increasing attention by the medical-imaging scientific community (Esteva et al., 2019).

Recent works uncovered the deep-learning potential to perform automatic US image-analysis tasks, including detection, classification, segmentation, biometric measurements, registration, and quality assessment, as well as emerging tasks such as image-guided interventions and therapy (Anas et al., 2015). It was also successfully applied, in medical US imaging analysis to different anatomical structures: breast (Bian et al., 2017; Hiramatsu et al., 2017), thyroid (Ma et al., 2017), heart/cardiac (Ghesu et al., 2016; Pereira et al., 2017), brain (Milletari et al., 2015; Sombune et al., 2017), fetus (Yaqub et al., 2017), and many other organs and body parts (see Liu et al., 2019 for a review). However, only a limited amount of studies investigated the performances of deep neural networks on lung ultrasound images, and these studies mainly focus on detecting and extracting domain specific, hand-crafted features such as A-lines, B-lines (also known as vertical artifacts), pleural lines (Carrer et al., 2020), pleural effusions, and consolidations, with B-line detection being the most common task (Kulhare et al., 2018; Wang et al., 2019; van Sloun and Demi, 2020).

The recent outbreak of the novel 2019 coronavirus (COVID-19) has required the development of fast diagnostic techniques, among which the chest X-ray is key. Given the paucity of radiologists and expertise in the field, in order to provide physicians with valid assistance for accurate diagnosis, there is an increased interest in quickly developing AI systems. Recently, there have been many publications that focused on using deep neural networks over raw chest X-ray images rather than learning hand-engineered features by using deep neural networks.

For instance, Wang et al. (2020) designed COVID-Net, an open-source deep convolutional neural network that was specifically tailored for the task of detecting COVID-19 cases from chest X-ray images. The authors also introduced COVIDx, an open-access benchmark data set that comprises 13,975 chest X-ray images across 13,870 patient cases. The COVID-Net achieves 93.3% test accuracy in classifying images from individuals who belong to three different classes: healthy, non-COVID pneumonia, and COVID-19.

Another example is the study from Apostolopoulos and Mpesiana (Apostolopoulos and Mpesiana, 2020) who applied transfer learning on state-of-the-art CNN architectures for classification tasks that involve COVID-19 as one of the target classes. Specifically, they used a database of chest X-ray scans that contain 224 images of patients with COVID-19, 700 images of people infected with non-COVID pneumonia, and 504 images of healthy individuals. The best performance model (VGG19) achieved an accuracy of 98.75% in classifying the following multi-class problem: normal vs. COVID-19 pneumonia vs. bacterial pneumonia. Whereas, an accuracy of 93.48% was reached for the multi-class problem: normal vs. COVID-19 pneumonia vs. viral and bacterial pneumonia.

All the above mentioned studies used deep learning and raw chest X-ray images for either binary (normal vs. COVID-19) or 3-class (normal vs. pneumonia vs. COVID-19) classification problems. These studies provided evidence that deep neural networks can achieve impressively high performance when applied to lung medical imaging without the need for explicitly designed and extracted problem-oriented features to be fed into the neural network. Therefore, these works confirm the idea that deep-learning techniques have the potential to change the design paradigm of the computer-aided diagnostics (CADx) systems (Bian et al., 2017) and provide physicians with refined interpretations of medical imaging (McBee et al., 2018).

Less attention has been given to the use of deep learning for the automation of lung disease classification from raw ultrasound images. Nonetheless, LUS has been shown to play an invaluable role in the diagnosis, management, and prognosis of COVID-19 in all age groups (Bonadia et al., 2020; Buonsenso et al., 2020a; Smith et al., 2020; Volpicelli et al., 2020, 2021) including children (Musolino et al., 2021) and pregnant women (Buonsenso et al., 2020b; Inchingolo et al., 2020). Remarkably, LUS has a diagnostic accuracy similar to that of chest X-ray in COVID-19 patients (Lieveld et al., 2020; Pare et al., 2020; Tung-Chen et al., 2020).

Only recently, Born et al. (2021) presented a VGG16-based CNN, POCOVID-Net, pre-trained on ImageNet (Deng et al., 2009), and then fine-tuned it by using their data set, the largest publicly available LUS data set for COVID-19: 1,204 COVID-19, 704 bacterial pneumonia, and 1,326 healthy images. Their convolutional neural network was able to differentiate among patients who were diagnosed with COVID-19, those who were affected by bacterial pneumonia, and healthy individuals, thus achieving an overall accuracy of 89%.

Inspired by the above research results on COVID-19, obtained by applying deep-learning techniques to raw medical imaging, we analyse the performance of different state-of-the-art convolutional neural networks for the diagnosis of childhood pulmonary disease. In this first study we included a large set of LUS images from children with lower-respiratory-tract infections; this represents a real practice scenario in the pediatrics department, for children affected by the most important pediatric respiratory conditions (pneumonia and bronchiolitis), as well as healthy subjects.

In order to achieve automatic feature extraction, we applied deep-learning techniques directly to raw LUS images. For this purpose, we trained from scratch the following state-of-the-art deep-learning models: VGG19, Xception, Inception-v3, and Inception-ResNet-v2. In this work, we assess the performance of one binary classification problem and one three-class classification problem, including LUS images of healthy infants, those with bronchiolitis, and those with bacterial pneumonia.

## 2. Methods

### 2.1. Participants

For the purpose of this study, we considered three group of patients enrolled in clinical studies at the Agostino Gemelli University Hospital between the end of 2018 and the beginning of 2019:

- Healthy: 33 healthy infants (18 males, 15 females; mean age: 2.83 ± 2.89 months)
- Bronchiolitis: 22 infants with bronchiolitis (13 males, 9 females; mean age: 2.78 ± 2.96 months)
- Bacterial pneumonia: 7 children with bacterial pneumonia (4 males, 3 females; mean age: 7 years ± 6.85 years).

Healthy infants were defined as children completely healthy without comorbidities.

Infants with bronchiolitis were diagnosed through an integrated approach based on clinical (Seattle Children's Hospital, 2011) and ultrasound assessment (Buonsenso et al., 2019b; Supino et al., 2019). Radiological evaluation and laboratory tests (e.g., oxygen saturation) were also performed, when necessary, as recommended by clinical practice guidelines (Subcommittee on Diagnosis and Management of Bronchiolitis, 2006; Ralston et al., 2014). All patients suspected to have bronchiolitis underwent a routine clinical assessment based on Seattle Children's Hospital clinical scores (Seattle Children's Hospital, 2011). This is a clinical score created by Seattle Children's Hospital in 2011, to evaluate children with bronchiolitis and to distinguish those in need of hospitalization from those who could be discharged. The score is based on the consideration of

- respiratory rate
- retractions: subcostal, intercostal, or supraclavicular retractions; nasal flaring; or bobbing of the head
- signs of dyspnea: reduction/suspension of feeding, reduction/suspension of vocalization, agitation, drowsiness, or confusion
- auscultation: inspiratory wheeze, expiratory wheeze, or reduction of air penetration.

The clinical diagnosis of CAP was made in accordance with the British Thoracic Society guidelines (Harris et al., 2011). At the first evaluation, all children with suspected CAP underwent: medical history, clinical evaluation, and blood tests, including complete blood count (CBC) with white blood cell (WBC), and C-reactive protein (CRP). Definitive diagnoses of bacterial pneumonia were confirmed by both chest X-ray and lung ultrasound.

Only the children that underwent complete LUS scanning as described below, have been included in the analyses. Images from each patient were collected during multiple sessions, therefore they display a disease with different severity in the same patient. Images and videos have been collected during the study protocol “Utility of lung ultrasound in children with lower respiratory tract infections”, approved by the Ethic Committee of the Fondazione Policlinico Universitario A. Gemelli IRCCS, Rome, Italy (prot 36173/19, ID 2729). Written informed consent was obtained before data collection by the caregiver of the study participants. All the private information of patients was anonymized.

### 2.2. Lung Scanning Procedure

LUS imaging was performed with the ultrasound machine ESAOTE MyLab™ 40 using a linear probe (12–6 MHz). Images and clips were stored and archived. In order to guarantee agreement in the methodology and acquisition, all LUS scanning were performed by two physicians, Danilo Buonsenso and Cristina De Rose, with more than 5 years of experience in LUS clinical practice and teaching and already several papers published together (Buonsenso et al., 2020c,d; Pata et al., 2020; Rose et al., 2020). The scans were made by investigating the anterior, lateral, and posterior regions of the thorax, according to a protocol used by the Italian Academy of Thoracic Ultrasound (ADET) and recently published in a COVID-19 protocol (Taccari and Buonsenso, 2020).

The following lung ultrasound features were evaluated to better define the prognosis of the infants with bronchiolitis and, in particular, to identify those children who are in need of supplementary oxygen (Basile et al., 2015; Taveira et al., 2018; Buonsenso et al., 2019b; Supino et al., 2019):

- presence of an irregular pleural line;
- absence of pleural effusion;
- presence of short vertical artifacts;
- presence of long vertical artifacts: multiple, non-confluent, and/or confluent, unevenly distributed, possibly involving several lung areas, possibly bilaterally distributed, and with “spared areas” in the single area involved;
- presence of area(s) of white-lung;
- presence of single or multiple subpleural consolidations (even > 1 cm in size), associated with multiple long non-confluent or confluent vertical perilesional artifacts.

Lung ultrasound features considered for the aetiological diagnosis of bacterial pneumonia were as follows (Berce et al., 2019; Buonsenso et al., 2021):

- irregular pleural line;
- subpleural pulmonary parenchymal lesion (consolidation and atelectasis; > 2 cm, and in particular > 4 cm);
- presence of bronchograms, its characteristics (air or fluid), morphology (arboriform or dot-like/linear), position (deep if > 2 cm far from the pleura or superficial if close to the pleura), dynamicity during breath (fix, poorly dynamic, or clearly dynamic);
- presence and type of pleural effusion: simple (anechogenic and dependent on gravity) or complex (presence of septa, hyperechogenic spot, following the lung through the apex and not dependent on gravity, requiring drainage).

We also report the lung ultrasound features that were evaluated for healthy infants, especially in the first 3 months of life (Buonsenso et al., 2020d):

- absence of irregularities of the pleural line;
- absence of pleural effusion;
- absence of subpleural consolidations;
- presence of short vertical artifacts;
- presence of long vertical artifacts single and/or multiple, non-confluent and/or confluent, with possible uneven distribution and/or involving multiple lung areas with a prevalence of the right and/or left hemithorax, depending on the gestational age and the current age of the patient.

### 2.3. Data Acquisition

The set of available lung ultrasound images were ordered by the patients and manually categorized by medical operators into three different diagnoses: healthy infants, infants with bronchiolitis, and children with bacterial pneumonia. The resulting data set of all available images is organized as follows:

1. Healthy: 5,907 images: 5,193 bmp images validated by human raters and 714 bmp images automatically extracted from videos and validated by human raters.
2. Bronchiolitis: 3,286 images: 2,516 bmp images validated by human raters and 770 automatically extracted from videos and validated by human raters.
3. Bacterial pneumonia: 4,769 images: 206 bmp images validated by human raters and 4,563 automatically extracted from videos and validated by human raters.

Every patient contributes to multiple ultrasound images (mean = 226.1 ± 287.4), with a minimum of 43 images, collectively taken from different sessions. To estimate the accuracy of the classifiers, we used four-fold cross-validation stratified by the number of samples per class. In order to avoid the unbalanced data problem, we sampled images from the original data set of available images as follows. Data were split on a patient level, hence we organized the folds in such a way that all the images belonging to a particular subject were assigned to only one-fold and, consequently to only one of the following subsets: training set, validation set, test set (i.e., there was no overlap patient-wise among the training, validation, and test data sets). Where possible, the subset of patients was matched by age and gender, and it was distributed randomly between the folds as per the above mentioned constraints. Each of the four final data sets used for four-fold cross-validation is organized as follows:

1. Healthy: 2,000 images: 1,000 bmp images for the training set, 500 images for the validation set and 500 images for the test set.
2. Bronchiolitis: 2,000 images: 1,000 bmp images for the training set, 500 images for the validation set and 500 images for the test set.
3. Bacterial pneumonia: 2,000 images: 1,000 bmp images for the training set, 500 images for the validation set and 500 images for the test set.

### 2.4. Convolutional Neural Networks

Deep learning constitutes the state-of-the-art set of applied machine-learning techniques and frameworks currently used both in the research and industry fields to perform automated tasks such as signal classification, regression, image segmentation. Such frameworks are also currently used in the medical domain to draw meaningful results from medical data: for instance, the automated classification or segmentation of magnetic resonance imaging (MRI), computed tomography (CT), and X-ray images.

One fundamental class of deep neural networks, applied mostly in analyzing visual imagery, is represented by convolutional neural networks (CNNs). In fact, CNNs are able to process data expressed as tensors, called feature maps, i.e., three-dimensional arrays. For instance, an RGB image is a 3D tensor with two spatial axes (height and width), as well as three depth axes (also called the channels axis). Each depth channel accounts for a single color component: red, green, or blue.

The typical CNN architecture is structured as a series of stages. The fundamental data structure being the layer: a data processing module that takes one or more tensors as input and returns one or more tensors as outputs. Most of deep learning consists of chaining together simple layers that will implement a form of progressive information distillation over the input data: a succession of increasingly refined data filters are applied by going deeper in the CNNs. These layers can either be stateless or have a state; the weights are the state of the layers. Weights are tensors learned with stochastic gradient descent and, collectively, they constitute the knowledge of a neural network. CNNs are usually constructed making use of different types of layers: convolutional layers, pooling layers, fully connected layers, and others.

The feature extraction process takes place in both convolutional and pooling layers, whereas the classification process occurs in the fully connected layer. It is important to note that the topology of a neural network defines a hypothesis space for the target distribution, i.e., the distribution over which the final system performance must be trained. In fact, machine learning (hence deep learning) accomplishes the task of looking for useful representations of some desired distribution of data, within a predefined space of possibilities, by using a feedback signal as search guidance, i.e., backpropagating gradients through the CNNs. Every time a network topology is chosen, the space of possible hypotheses is constrained in some way: specifically a series of tensor operations are chosen to be used for mapping input data to output data. Training a neural network means finding a good set of values for the weight tensors involved in the tensor operations that map inputs into outputs thus enabling a single model (or hypothesis) for the target data distribution to be selected.

#### 2.4.1. Convolutional Layer

The convolutional layer is the base layer of a CNN. In a convolutional layer, during the inference process, patches from the layer input feature map are extracted and transformed into output feature map (response map) by applying the same convolution operation to each patch.

The output feature map is a 3D tensor with the width, height, and an arbitrary number of depth channels. Every channel in the depth axis stands for a filter and the response map is a 2D tensor that indicates the response of the filter over the input. Filters encode specific aspects of the input data, i.e., features. As the activation map is obtained by performing convolution between the filter and the input, the filter parameters are spatially invariant. Therefore, CNNs are particularly efficient when processing images: as the visual world translation is invariant, only a limited amount of training data is needed to learn representations with great generalization ability. CNNs can also learn spatial hierarchies of patterns: the first convolutional layer will learn small local patterns such as edges; a second convolutional layer will learn larger patterns with features of the first layers as building blocks, and so on.

#### 2.4.2. Pooling Layer

Usually, a pooling layer follows a convolutional layer. The pooling layer is applied to reduce the spatial dependency of the computed features maps, hence to increase robustness to changes in the position of the feature in the image and to better exploit the resulting feature hierarchy. This is achieved by downsampling the feature maps in order to keep them reasonable in number.

The downsampling can be performed through the use of different techniques such as max-pooling and average pooling. The max-pooling operation, similarly to the convolution operation, extracts local patches from the input feature maps and outputs the maximum value of each channel in the original visual patch. The average pooling, instead, outputs the average value of each channel over the patch. Max-pooling tends to perform better than average pooling, as the maximal presence of specific features are more informative than their average presence.

#### 2.4.3. Fully Connected Layer

Fully connected layers are final layers in a CNN where each neuron is completely connected to other neurons. These layers are responsible for the final classification results. In a fully connected layer, the rectified linear unit (ReLU) activation function is commonly used:

(1) $$\begin{array}{l} ReLU\left(x\right)=\{\begin{array}{cc} 0, & x<0\\ x, & x\geq 0 \end{array} \end{array}$$

Softmax activation function is usually utilized to predict output images in the very last fully connected layer:

(2) $$\begin{array}{l} Softmax\left(x_{i}\right)=\frac{e^{x_{i}}}{\sum_{y=1}^{m}e^{x_{y}}} \end{array}$$

where *x*_*i*_ and *m* represent input data and the number of classes, respectively.

#### 2.4.4. Hyper-Parameter Optimization

Deep-neural-network performance depends on a wide range of hyper-parameter choices, subject to fine tuning during the training process and include, among others, the CNN architecture, an optimization process, and regularization (Hutter et al., 2019).

A CNN optimization configuration involves selecting the optimizer to be used to update the network weights through stochastic gradient descent. The learning rate of the optimizer defines the magnitude of the modifications to the model weights in response to the estimated error. By applying a learning rate that changes during training (i.e., adaptive learning rate), increased performance and a faster convergence can often be achieved. For instance, a learning-rate decay formula might be used to reduce the learning rate at each iteration *i* (e.g., end of each mini-batch) as follows:

(3) $$\begin{array}{l} learning\text{\_}rate_{i}=learning\text{\_}rate_{i-1}*\left(\frac{1}{1+decay*\left(i-1\right)}\right) \end{array}$$

Regularization is a design principle for augmenting a primary optimization objective (e.g., how well a learned model fits its training data) by taking into account a secondary objective: a penalization term with respect to those representations that are less desirable due to less compact. In weight regularization, for example, a cost is associated with the loss function of the network in order to constrain the CNN weight values to be small and the distribution of weight values to be regular. The cost might be proportional to either the absolute value of the weight coefficients (L1-regularization) or to the squared value of the weight coefficients (L2-regularization). The dropout regularization technique consists of introducing noise in the output values of a layer by randomly setting a fraction of them to zero, i.e., dropping them out, during the training phase. The idea behind this is to prevent the CNN from retaining the patterns that are less significant.

### 2.5. Experimented CNN Architectures

CNN architectures are crafted by stacking different types of layers and can result in networks that have very deep structures. We here present an overview of some of the most relevant, in literature, existing CNN architectures that have been trained and tested in this experimental investigation.

#### 2.5.1. VGG19

The VGG19 CNN architecture (Simonyan and Zisserman, 2015) was introduced in 2014, as an improvement of the well-known VGG16. The main contribution resulted in an increased depth of the network and by the replacement of the 11 × 11 and 5 × 5 with small 3 × 3 convolutional filters. The network consists of 19 layers (16 convolutional layers, 3 fully connected layers, 5 max-pooling layers and 1 Softmax layer). The default input image size of VGG19 is 224 × 224 pixels. VGG19 showed a significant improvement on classification tasks with respect to the ImageNet Challenge 2014 and compared to other popular networks such as AlexNet and GoogleNet.

#### 2.5.2. Inception-v3

Inception CNN was introduced by Szegedy et al. (2016) at Google in 2013–2014. This is a popular CNN architecture, aimed at reaching performance efficiency by utilizing suitably factorized convolutions and aggressive regularization (see section 2.4.4). Factorized convolutions are effectively applied in CNN convolutional layers to simultaneously perform spatial convolution in each channel and linear projection across channels. These and other techniques can effectively preserve the spatial information and maintain the accuracy with significantly less computation (Wang et al., 2017). The default input image size of Inception-v3 is 299 × 299 pixels. The network input is processed by several parallel convolutional branches that work independently and whose outputs are then merged back into a single tensor. The most basic form of an Inception module has three to four branches that start with a 1 × 1 convolution, are followed by a 3 × 3 convolution, and end with the concatenation of the resulting features. This structure enables the network to learn, separately rather than jointly, spatial features and channel-wise features. The rationale behind this approach is the fact that each channel might be highly autocorrelated across space, but might not be highly correlated with other channels.

#### 2.5.3. Xception

Xception (Chollet, 2017) is a CNN architecture roughly inspired by Inception. Xception stands for extreme Inception. In fact, it adopts an extreme form of an Inception module: the process of learning channel-wise features is fully separated from that of learning spatial features. Moreover, the Xception network substitutes Inception modules with depth wise separable convolutions. They are depth wise convolutions (a spatial convolution where every input channel is handled separately) followed by a point-wise convolution (a 1 × 1 convolution). Xception and Inception-v3 have approximately the same number of parameters and the same default image size. However, Xception makes a more efficient use of model parameters with respect to Inception; therefore, it shows better runtime performance and higher accuracy large-scale data sets such as on ImageNet.

#### 2.5.4. Inception-ResNet-v2

Inception-ResNet-v2 (Szegedy et al., 2017) is a CNN architecture that belongs to the Inception CNN family and incorporates residual connection as a replacement of the filter concatenation stage of the Inception architecture. A residual connection resides in reintroducing previous representations by skipping one or more layers (through the so-called “shortcut connections”) and by summing by a past output tensor to a later output tensor. This helps to prevent information loss along the data-processing flow. The authors reported, on the one hand, a significant improvement of the recognition performance but, on the other, a substantial increase of the training speed compared to standard for the Inception architectures. The default input size for this model is 299 × 299 pixels.

### 2.6. Data Pre-processing

All ultrasound images were read into an RGB format to ensure that the model input shape is compatible with the used CNN models. Images containing artifacts, such as calipers, text, lines, and tick marks, were not considered in the current analysis. In fact, such patterns are detrimental for accurate image classification. Therefore, we used a simple template-matching module to detect and discard, prior to feeding the data set into the learning architecture, the ultrasound images that have these structures. The remaining images were cropped to remove uninformative data, such as dark borders and text, thus resulting in images with a resolution that span between 546 × 410 and 175 × 409 pixels. All images were then resized to the default input size of the used neural network (224 × 224 pixels for VGG19; 299 × 299 pixels for Inception-v3, Xception, and Inception-ResNet-v2) and were normalized to ensure every pixel value is between −1 and 1.

### 2.7. Data Augmentation

In order to avoid overfitting, we applied the technique of data augmentation (Perez and Wang, 2017) on the three-class classification problem. Data augmentation consists of artificially increasing the number of existing samples, by applying a number of random transformations: this yields close-to-real biomedical images that are likely to well represent the target data distribution.

The performance of different affine transformations were evaluated: flips (horizontal, vertical), angle rotations, translational pixel shifts, regional zoom, random Gaussian noise, and blurring by various amounts.

We found that the best performances are achieved by using a horizontal flip and width shift range of 10%. In fact, these transformations provide realistic lung ultrasound images. Horizontal flip produces horizontally-mirrored images of the lung, which might represent the occurrence of the clinical condition displayed in the original image but in the opposite lung. Similarly, a width shift range of 10% can represent a lung from a slightly older patient. The images were expanded from 3,000 lung ultrasound images to 100,000 artificial images. It should be noted that augmentation was only done for the training data set; the validation and the testing data sets were not touched.

### 2.8. Experimental Setup

Keras (Chollet, 2015), a compact, high-level and easy-to-learn Python library for deep learning, coupled with TensorFlow backend (Abadi et al., 2016) in Python 3.7 was used to train the deep-learning models from scratch.

Several python libraries [Qt (Nokia Corp., 2012), OpenCV (Bradski, 2000), Sklearn (Buitinck et al., 2013)] were used for the statistical analysis and the software implementation, including the development of a software system that uses the learned model for classifying images of healthy children and those with either bronchiolitis or pneumonia.

All experiments were performed on a workstation Intel^Ⓡ^ Xenon^Ⓡ^ CPU E5-2680 @ 2.70 GHz (2 processors) with Windows 10 operating system using GeForce RTX^*TM*^ 2080 Ti GPU graphics card.

### 2.9. Model Selection

In this section, we provide a brief description of the CNNs employed for LUS images classification. All CNN models (VGG19, Xception, Inception-v3 and Inception-ResNet-v2) were trained from scratch with random initialization weights. The default densely connected classifier (from ImageNet) on top of the network, for all Keras models, were replaced with new fully-connected layers having the correct number of output classes.

The trained neural networks share some common hyper-parameters. More specifically, all CNNs were compiled using the optimization method called RMSProp, a momentum (Sutskever et al., 2013) with a decay of 0.9; and all the convolutional layers were activated by the rectified linear unit (ReLU). In all our experiments, we trained all the CNNs with batch sizes of 20 for 50 epochs, except for Inception-ResNet-v2 that was trained with a batch size of 10.

As for the three-class classification problems, we performed a hyper-parameter optimization (see section 2.4.4) with respect the following parameters:

- the learning rate was experimentally iterated between the value 1e-3 and value 1e-7: 1e-6 was selected;
- the dropout regularization was set to 0.5 in all experiments;
- l2-regularization: the following values have been iteratively evaluated 5e-4, 4e-5, 1e-5, 0: 0 was selected;
- exponential learning rate decay was set to either 0.94 or 0: 0 was selected.

### 2.10. Performance Metrics

Five criteria were used for evaluating the performances of deep-learning models.

(4) $$\begin{array}{l} Accuracy=\left(TN+TP\right)/\left(TN+TP+FN+FP\right) \end{array}$$

Accuracy is the ratio of the number of true samples to the total number of samples.

(5) $$\begin{array}{l} Sensitivity=TP/\left(TP+FN\right) \end{array}$$

Sensitivity, also known as true positive rate (TPR) or recall, is the probability that a patient with a certain condition is correctly diagnosed.

(6) $$\begin{array}{l} Specificity=TN/\left(TN+FP\right) \end{array}$$

Specificity is the probability that people without a certain diagnosis are not erroneously diagnosed as suffering from that disease. It can however analogously be called true-negative rate. Precision, also known as true positive accuracy (TPA), is defined in (7):

(7) $$\begin{array}{l} Precision=TP/\left(TP+FP\right) \end{array}$$

Precision denotes the proportion of positive predicted values (PPV) that are correctly real positives.

(8) $$\begin{array}{l} F1-Score=2*\left(\left(Precision*Recall\right)/\left(Precision+Recall\right)\right) \end{array}$$

The F1-Score is a measure of the accuracy of a model on a data set. This is defined as the harmonic mean of precision and recall. TP, FP, TN, and FN given in Equations (4–8) represent the number of true-positives, false-positives, true-negatives, and false-negatives, respectively.

The terms positive and negative are used to refer to the presence or absence of a condition of interest: bacterial pneumonia, bronchiolitis, or being healthy. True-positives (TP) are the number of examples correctly labeled as positives. False-positives (FP) refer to the number of samples incorrectly labeled as positive. False-positives (FP) refer to negative examples incorrectly labeled as positive. True-negatives (TN) correspond to negatives correctly labeled as negative. Finally, false-negatives (FN) refer to positive examples incorrectly labeled as negative.

## 3. Results

### 3.1. Experimental Results

In this paper, we performed one binary classification (healthy vs. bronchiolitis) and one three-class classification (healthy vs. bronchiolitis vs. bacterial pneumonia) out of three classes. The four-fold cross-validation method was used with four state-of-the-art deep learning models (Inception-v3, Inception-ResNet-v2, Xception, VGG19) trained from scratch. Fifty percent of the data is reserved for the training set, 25% of the data is allocated to the validation set, and the remaining 25% is reserved for the testing test. The experiments were repeated four times, until each 25%-part of the original data set was tested.

The results of all the experiments are listed in Table 1: it shows a detailed comparison of all trained models in terms of precision, sensitivity, F1-scores, specificity, and accuracy for each fold; the average classification performances of the model were also calculated.

**Table 1:** Classification metrics.

**Healthy vs. Bronchiolitis**					
	**Precision**	**Sensitivity**	**F1-score**	**Specificity**	**Accuracy**
**Inception-v3**					
Fold 1	92	91	91	100	91
Fold 2	95	95	95	95.4	95
Fold 3	99	99	99	97.2	99
Fold 4	91	89	89	79.2	89
Average	94.25	93.5	92.95	92.95	93.5
**Inception-ResNet-v2**					
Fold 1	99	99	99	99.2	99
Fold 2	98	97	97	97.2	97
Fold 3	99	99	99	99	99
Fold 4	97	96	96	92.6	96
Average	98.25	97.75	97.75	97	97.75
**Xception**					
Fold 1	92	91	91	100	91
Fold 2	97	97	97	100	97
Fold 3	99	99	99	100	99
Fold 4	95	94	94	94	94
Average	95.75	95.25	95.25	98.5	95.25
**VGG19**					
Fold 1	90	89	88	89	94
Fold 2	92	92	92	95.2	92
Fold 3	96	96	96	95	96
Fold 4	89	87	87	74.8	87
Average	91.75	91	90.75	88.5	92.25
**Healthy vs. Bronchiolitis vs. Bacterial Pneumonia**					
	**Precision**	**Sensitivity**	**F1-score**	**Specificity**	**Accuracy**
**Inception-v3**					
Fold 1	92	92	92	88.68	92
Fold 2	92	91	91	99.75	91
Fold 3	94	92	92	99.79	92
Fold 4	92	91	91	95.26	91
Average	92.5	91.5	91.5	95.86	91.5
**Inception-ResNet-v2**					
Fold 1	96	96	96	98.01	96
Fold 2	90	87	87	99.69	87
Fold 3	88	82	81	100	82
Fold 4	88	82	81	100	82
Average	90.5	86.75	86.25	99.42	86.75
**Xception**					
Fold 1	96	96	96	93.97	96
Fold 2	89	83	82	100	83
Fold 3	82	68	61	100	68
Fold 4	95	95	95	95.45	95
Average	90.5	85.5	83.5	97.35	85.5
**VGG19**					
Fold 1	91	90	90	81.11	90
Fold 2	90	89	89	94.01	89
Fold 3	96	96	96	93.49	96
Fold 4	89	87	87	87.79	87
Average	91.5	90.5	90.5	89.1	90.5

For experiments classifying healthy vs bronchiolitis we found that the Inception-ResNet-v2 model provided the best results with a sensitivity of 97.75%, an accuracy of 97.75%, and a precision of 98.25%. Inception-v3 and Xception had similar performances. VGG19 was the worst and achieved accuracy of only 92.25%, a sensitivity of 91%, and a precision of 91.75%.

For experiments classifying healthy vs bronchiolitis vs bacterial pneumonia, we found that the Inception-v3 model provided the best results with a sensitivity of 91.5%, a precision of 92.5%, and an accuracy of 91.5%.

**Figure 1:**
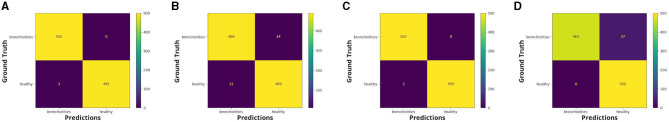
Confusion matrices: healthy vs. bronchiolitis, respectively for **(A)** fold 1, **(B)** fold 2, **(C)** fold 3, and **(D)** fold 4.

**Figure 2:**
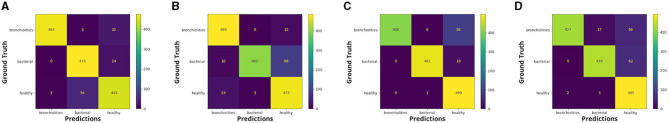
Confusion matrices: healthy vs. bacterial pneumonia vs. bronchiolitis, respectively for **(A)** fold 1, **(B)** fold 2, **(C)** fold 3, and **(D)** fold 4.

The confusion matrices in Figures 1, 2 report the number of TP, TN, FP, and FN results of our experiments for each fold. We can observe that, for the comparison between healthy and bronchiolitis, the number of false-negative predictions (FN) and the number of false-positive predictions (FP) are very low.

In the medical context, and in particular for the diagnosis of bronchiolitis, the minimization of false-negative predictions is crucial because not identifying the disease could lead to treatment delay, hence to the aggravation of symptoms, and poor medical outcomes.

We see that for the comparison healthy vs bronchiolitis vs. bacterial pneumonia, false-positive predictions (FP) are higher for the groups of healthy infants and those with bronchiolitis. This is probably due to the fact that infants show artifactual patterns that are similar to those patterns usually observed in pulmonary diseases.

We can notice from the matrices that there are cases in which LUS images correctly classified in the binary classification problem were then attributed to the incorrect class in the three-class classification problem by the best performance model, Inception-v3.

### 3.2. Statistical Results

In order to analyse statistically significant differences among ages of the different diagnostic groups, we performed Kruskal-Wallis *H*-tests. According to the result of the Kruskal-Wallis *H*-test, age does not statistically differ between the group of infants with bronchiolitis and the healthy infants (chi-squared = 0.28869, df = 1, *p*-value = 0.5911 > 0.05). Instead, the age of the children with bacterial pneumonia differs significantly from the age of children in the other two groups: healthy and bronchiolitis (chi-squared = 20.559, df = 2, *p*-value = 3.433e-05 < 0.05).

### 3.3. EXplainable Artificial Intelligence Results

EXplainable Artificial Intelligence (XAI) is a newly emerging discipline of AI (Doran et al., 2017) that seeks to develop a series of ML techniques that enable non-expert audiences to better understand and manage results obtained by artificial intelligence (Holzinger et al., 2017). In fact, deep-learning models are usually perceived as “black boxes,” they receive an input and learn representations that are in general difficult to extract and to present in a human-intelligible form. Although this concept is partially valid for certain types of deep-learning models, this is definitely not true for CNNs. Indeed, CNNs learn representations of visual concepts hence are highly responsive to visualization. We present a visualization study performed by using the best performance model (Inception-v3) that classifies images as belonging to three different diagnostic groups (i.e., bronchiolitis, pneumonia, and healthy). We applied three state-of-the-art eXplainable Artificial Intelligence approaches that are specifically tailored for convolutional neural networks: visualization of CNN filters, visualization of activation maps, and visualization of gradient-weighted class activation mapping (Grad-CAM).

#### 3.3.1. Visualizing CNN Filters

We can describe a deep network as a multistage information-distillation operation (see section 2.4): the information goes through successive layers, it becomes increasingly purified over successive filtering operations and is finally more informative for the task at hand. In fact, deep convolutional models learn a collection of filters that are increasingly refined and complex, the deeper the layers become. Specifically, each layer in a CNN learns a collection of filters such that their inputs can be expressed as a combination of the filters.

Visualizing CNN filters enables us to understand precisely which visual patterns or concepts each filter in a CNN is receptive to, therefore it permits us to observe how CNN layers see the world.

**Figure 3:**
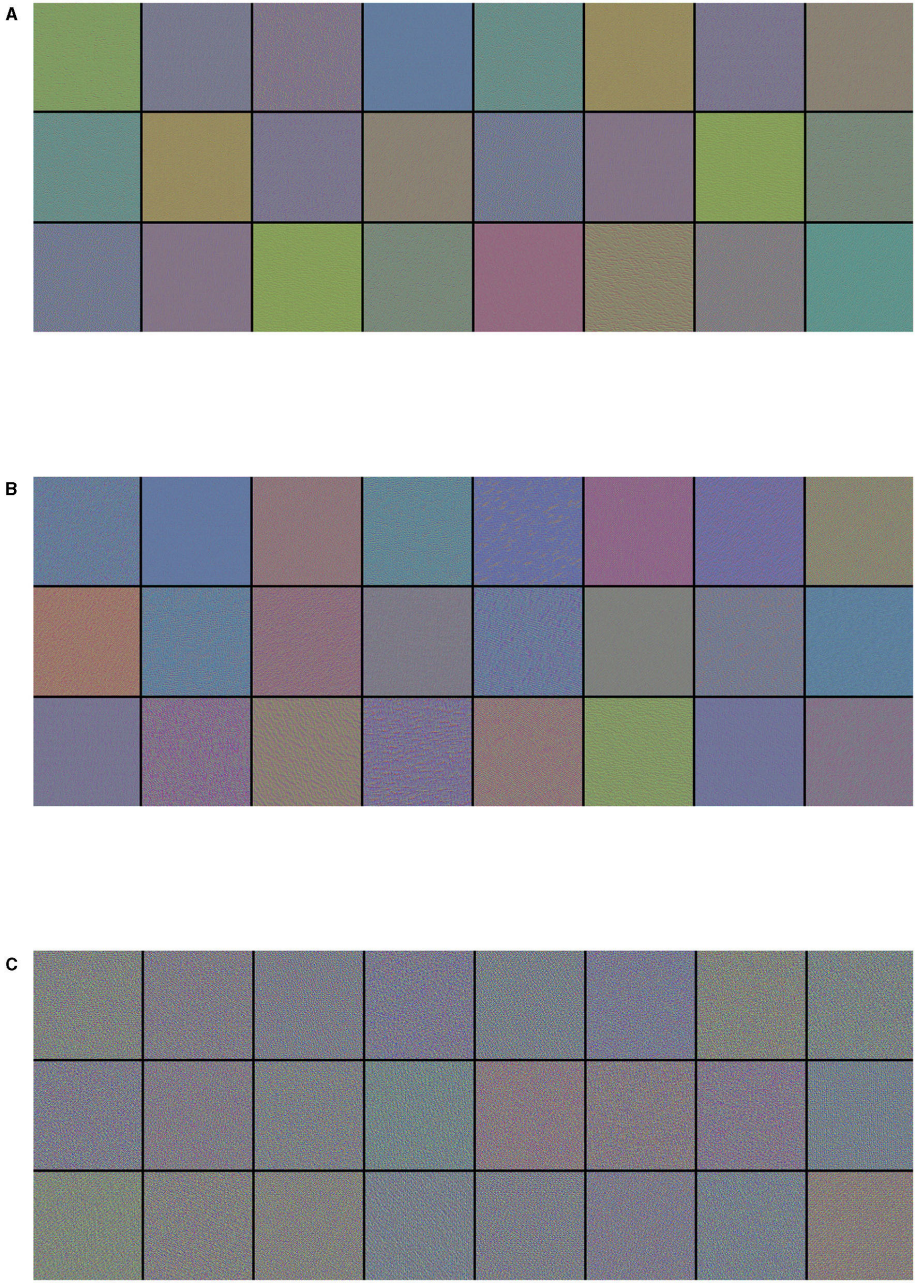
Visualization of **(A)** the first 24 filters from the first convolutional layer, **(B)** the first 24 filters from the third convolutional layer, and **(C)** the first 24 filters from the fifth convolutional layer of the best performance model (Inception-v3) on the three-class classification problem. Filters act as collections of edge detectors, detect background, contours, and texture-like patterns.

The filters from the first convolutional layers learned by the best performance model (Inception-v3) for the three-class classification problem are displayed in Figure 3. These filters encode colors, simple directional edges and, in some cases, colored edges that can be found in ultrasound images of lungs; the texture-like patterns of the filters become more complex, the deeper the layer becomes.

As a CNN is a hierarchical-modular network of convolutional filters that are probabilistically combined together, the way it works differs from the nature of human vision, which is not purely convolutional and it is organized in more sophisticated functionalities that involve motor control (Bressler, 1995). Nonetheless, colors, simple directional edges, and texture-like patterns extracted by a deep-learning model can provide useful insights that could help physicians diagnose lung diseases.

#### 3.3.2. Visualizing Intermediate Activations

**Figure 4:**
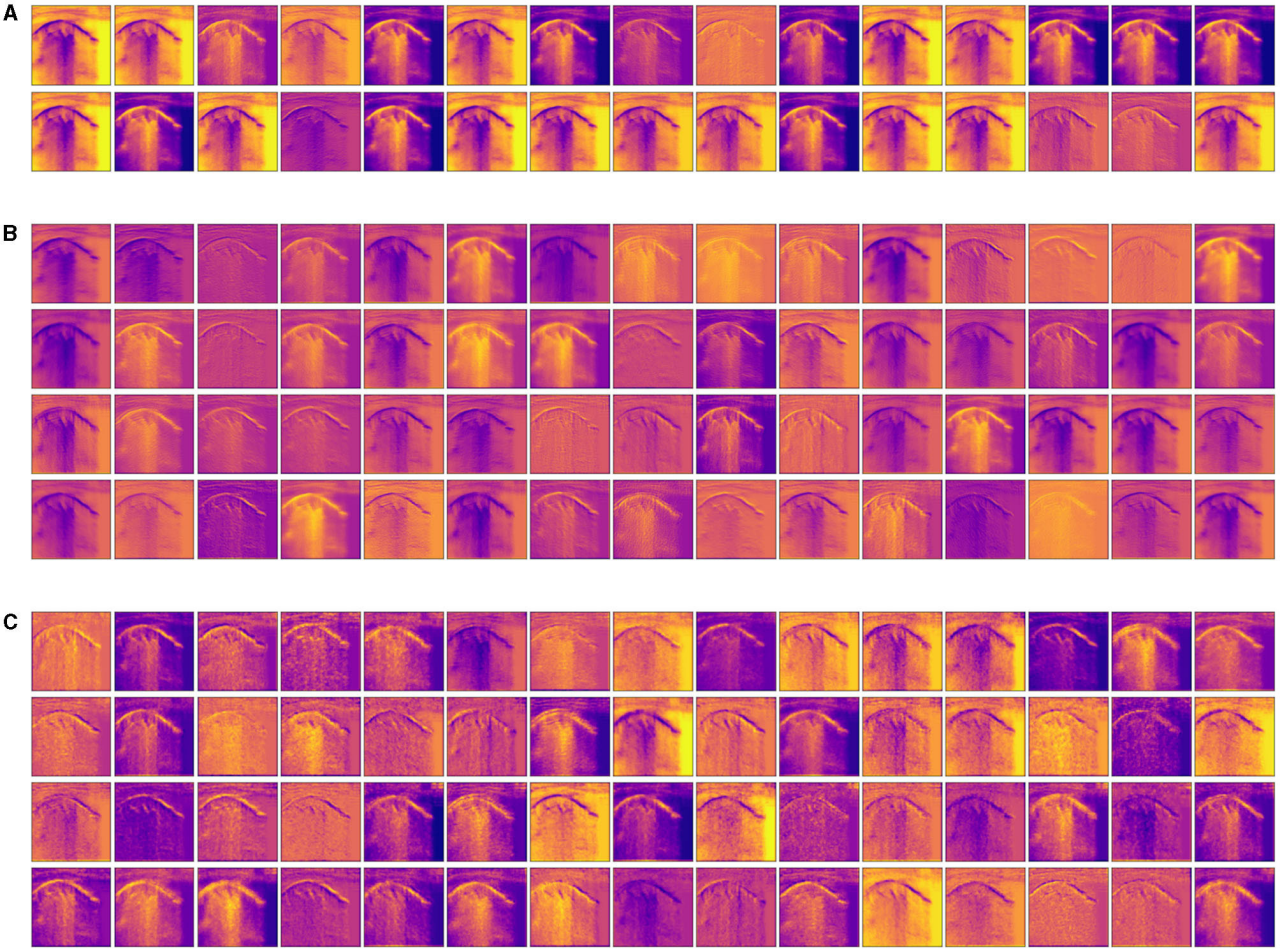
Visualization of the activation maps from **(A)** the first, **(B)** the third, and **(C)** the fourth convolutional layer of the best performance model (Inception-v3) on the three-class classification problem, when fed with an image of a lung with bronchiolitis. Filters act as collections of edge detectors, detect background, contours, and texture-like patterns. When going deeper in the layers, the filters enhance differently vertical artifacts and small consolidation-like patterns that are typical of bronchiolitis.

Visualizing intermediate activations (i.e., intermediate output of the activation function) consists of displaying, given a certain input, the feature maps that are output by various convolutions and by pooling layers in a network. Intermediate CNN outputs enable us to visualize the result of applying individual CNN filters to an input image, thus enabling us to visualize how an input is decomposed into the different filters learned by the network. In fact, feature maps are presence maps of learned visual concepts over a picture. Given as input an ultrasound image of a lung with bronchiolitis, in order to gain further insight into its performance and learned behavior, we show some visualizations of the activations from intermediate layers of the best performance model (Inception-v3) for the three-class classification problem. We observe in Figure 4, that different filters in the first convolutional layers activate distinct parts of the ultrasound image. Some filters act as collections of edge detectors; some other filters detect the background, and others detect the contours and texture-like patterns. It is evident how the activations have almost entirely preserved and split the information present in the original image. When going deeper in the layers, filters enhance in different ways the B-lines and small consolidation-like patterns that are typical of bronchiolitis. At the same time, the images become a little blurry, due to the max-pooling operations. As more and more pooling layers are introduced, the extracted features become increasingly abstract the deeper the layers become: this is an important and universal characteristic of the representations learned by deep neural networks. As explained in section 2.4, the deep neural network acts as an information distillation pipeline, where activations of the higher layers carry less information about the specific input being seen, and more information about the target (i.e., the class being learned: bronchiolitis, pneumonia, or healthy lung), thus helping the complete network to finally classify the image properly but without providing us with much visual information. The sparsity of the activations increases together with the depth of the network: in the last layers, many feature maps are blank, meaning that the pattern encoded by the filters is not present in the input image. The representation learned by the filter at this stage is much more abstract and not directly present in the original image. This resembles human perception: after observing a scene for a few seconds, humans remember which particular categories of objects were present in their field of view but cannot recall the exact appearance of those objects. Although these visual concepts might be different from how a human interprets images, they might be useful in helping physicians make diagnoses.

#### 3.3.3. Visualizing Class-Activation Mapping

This visualization technique can be used to shed light on the reason a CNN model decides that an ultrasound image belongs to a certain class of diagnosis. Class-activation mapping highlights the parts of an image that are identified by means of the learned model, thus, they show where in the picture the features that characterize a diagnosis are located. In our examples, the red/orange areas are considered by the model to output the class prediction: the brighter the red color is, the higher the probability of the predicted class of diagnosis is.

**Figure 5:**
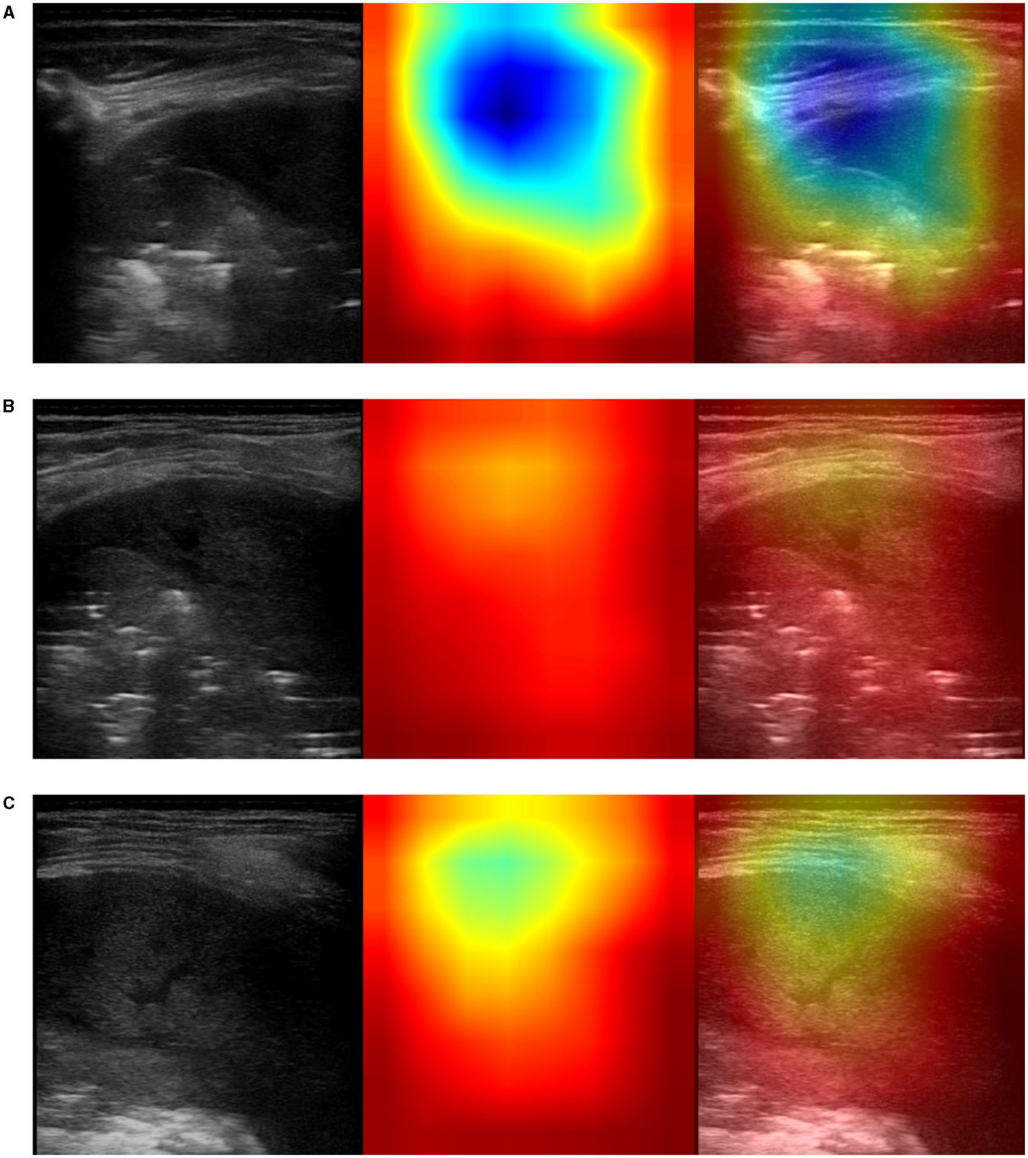
Example of lung ultrasound images correctly predicted by the best performance model (Inception-v3) as bacterial pneumonia. **Left**: original LUS image; **middle**: Grad-CAM visualization; **right**: the class activation mapping transparently overlaid on the original LUS image. Note that red and orange regions correspond to high scores for the predicted class and correctly highlight diagnostic-relevant features: **(A)** severe consolidations with fluid bronchogram and dynamic air bronchogram, **(B)** severe consolidations with fluid bronchogram and dynamic air bronchogram, complicated pleural effusions, **(C)** fibrinous pleural effusions with pulmonary atelectasis.

**Figure 6:**
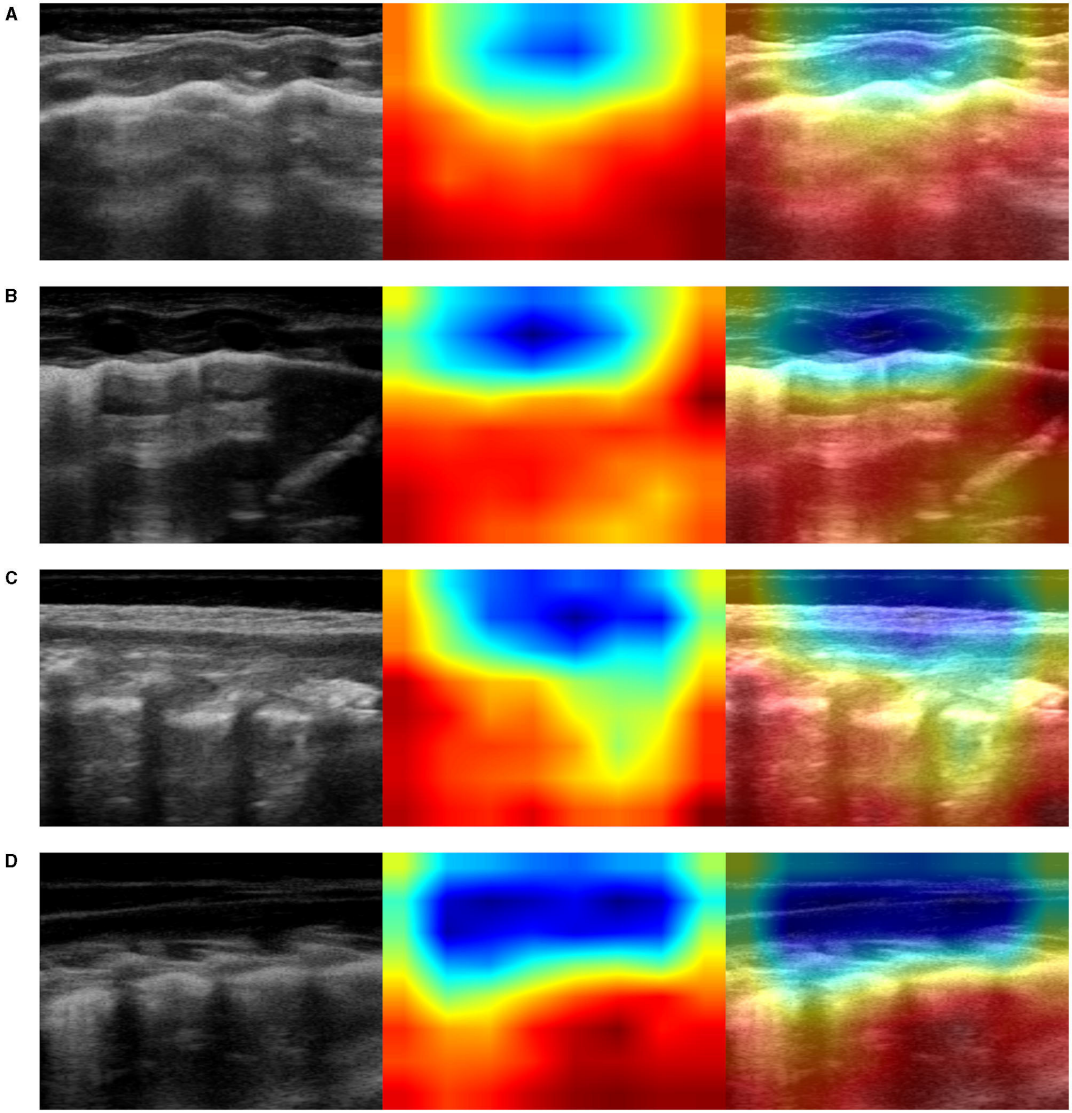
Example of lung ultrasound images correctly predicted by the best performance model (Inception-v3) as bronchiolitis. **Left**: original LUS image; **middle**: class activation mapping produced by the Grad-CAM visualization; **right**: the class activation mapping overlaid transparently on the original LUS image. Note that red and orange regions correspond to high scores for the predicted class and correctly highlight diagnostic-relevant features: **(A)** irregular short vertical artifacts; **(B)** irregular pleural line, subpleural consolidation > 2 cm, confluent short and long vertical artifacts; **(C)** irregular subpleural line, microconsolidations, short vertical artifacts; and **(D)** white-lung.

**Figure 7:**
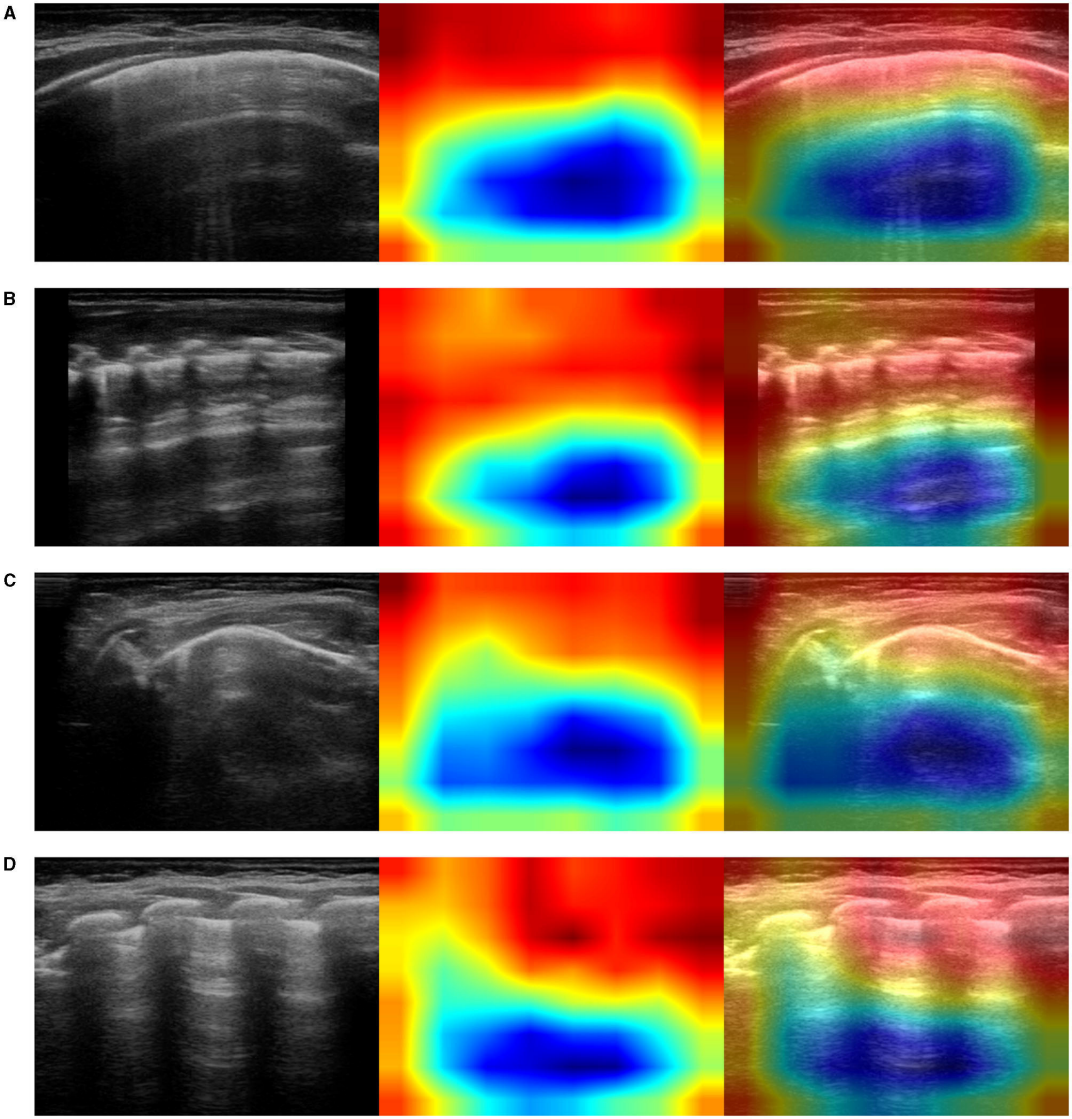
Examples of lung ultrasound images **(A–D)** from healthy infants correctly predicted by the best performance model (Inception-v3). **Left**: original LUS image; **middle**: class activation mapping produced by the Grad-CAM visualization; **right**: the class activation mapping overlaid transparently on the original LUS image. Note that red and orange regions correspond to high scores for the predicted class and correctly highlight diagnostic-relevant features: regular pleural lines; presence of short vertical artifacts; presence of long vertical artifacts single and/or multiple, non-confluent and/or confluent, with possible uneven distribution and/or involving multiple lung areas with a prevalence of the right and/or left hemithorax, depending on the gestational age and the current age of the patient.

In particular, we used the specific implementation that is described in “Grad-CAM: Visual Explanations from Deep Networks via Gradient-based Localization” (Selvaraju et al., 2017). We produced Grad-CAM visualizations for all LUS images belonging to the testing set and a set of corresponding images, each showing (1) the ultrasound images used as input to the optimal model, for the three-class classification problem, (2) the GRAD-Cam visualization, and (3) the overlapping of the lung ultrasound image and the class activation mapping. Examples of resulting images are shown in Figures 5–**7**.

The resulting images, together with the origin class and the predicted class by the optimal model, were qualitatively evaluated by a pediatrician expert in lung ultrasound. His comments are reported in section 4.

## 4. Discussion

### 4.1. Summary of Findings

To the best of our knowledge, this is the first study using deep-learning techniques and raw lung ultrasound images (LUS) for the purpose of diagnosing bronchiolitis and bacterial pneumonia in children. We trained from scratch state-of-the-art deep neural networks on a large data set - a training-set size of 2,000 LUS images for the binary classification problem. An initial training-set size of 3,000 LUS images was expanded with data augmentation to contain 100,000 artificially-created images for the three-class classification problem. We carried out comparisons with results from four different CNN networks trained using four-fold cross-validation: VGG19, Xception, Inception-v3, and Inception-ResNet-v2. The optimization of the supervised classifier was performed jointly with the optimization of the neural network.

We provide strong evidence that the automatic detection from lung ultrasound imaging, of pulmonary diseases in children, is a promising future research direction to be investigated. In particular, as shown in Table 1, we obtained high performance for the three-class classification problem involving healthy infants and those with cases of bronchiolitis and bacterial pneumonia: an average accuracy, sensitivity, and F1-score of 91.5%, and with precision and specificity, respectively, of 92.5 and 95.86%.

### 4.2. Clinical Significance

It is important to note that no hand-crafted features were considered in our algorithm pipeline. Nonetheless, biologically relevant features were automatically selected and extracted from the LUS images by the optimal deep-learning model. The Grad-CAM (see section 3.3.3), enables us to understand which areas in an image are mostly considered by the model to make its decision about the diagnosis. Dr. Buonsenso, a pediatrician expert in lung ultrasound, with more than 5 years of experience in LUS diagnosis and teaching, analyzed both the results of the classification by the optimal deep-learning model and the Grad-CAM visualizations. The purpose was to evaluate whether diagnostically relevant visual features in pulmonary diseases were highlighted by the Grad-CAM hence taken into consideration by the optimal deep-learning model for predicting the diagnosis. The model correctly identified almost all the images belonging to the group of children with bacterial pneumonia. The majority of the Grad-CAM also localized the domain-specific features that are taken into account by physicians when formulating a diagnosis of bacterial pneumonia: for example, larger consolidations with air and/or liquid bronchograms and pleural effusions, either simple (anaecogenic fluid) or complex (with fibrinae and septae, see Figure 5). Similarly, the model achieved high accuracy in classifying images belonging to infants with bronchiolitis. Consistently, the Grad-CAM detected diagnostically-relevant features (see Figure 6). In particular, in the case of bronchiolitis, the Grad-CAM often and suitably pointed out areas of short and long artifacts (either isolated or confluent), pleural line irregularities (with or without pleural effusions), large consolidations with air bronchograms, and small subpleural consolidations. The model showed the best performance when distinguishing larger consolidations and vertical artifacts, especially when they were long and confluent. Finally, the model showed great performance also in recognizing LUS images of healthy infants, even in presence of long vertical artifacts that might be characteristic of different conditions, see Figure 7.

### 4.3. Comparison With Other Studies

Few studies, for the purpose of assisting medical doctors make their diagnoses, have been dedicated to the investigation of lung diseases by applying deep-learning techniques to raw LUS images. For instance, Born et al. (2021) crafted a VGG16-based convolutional neural network pre-trained on ImageNet and successfully performed three-class classification problems involving cases of COVID-19 patients with bacterial pneumonia and healthy individuals. Following a different approach, we did not modify the structure of the original CNNs, except for the classifier. We also used random weights instead of those trained on ImageNet: lung ultrasound images are fundamentally different from those contained in the ImageNet data set, likely characterized by features that diverge from those identified with the use of the ImageNet data set.

Similar deep-learning approaches were extensively validated on CT scans and chest X-ray for COVID-19 diagnoses and were previously applied to pediatric pulmonary diseases by using chest X-ray images.

Ozturk et al. (2020) presented a deep neural network based on a DarkNet model for the automatic COVID-19 detection using chest X-ray images. They implemented 17 convolutional layers with LeakyReLU as activation function and introduced different filtering on each layer. The model achieved an accuracy of 98.08% for binary classes (normal vs. COVID-19) and 87.02% for multi-class cases (COVID-19 vs. no-findings vs. pneumonia). In work more comparable to our study, Narin et al. (2021) conducted experiments with three binary classification problems by using transfer learning on five pre-trained convolutional neural-network-based models (ResNet-50, ResNet-101, ResNet-152, Inception-v3, and Inception-ResNet-v2), and they tested their performance with five-fold cross-validation. They found that the pre-trained ResNet-50 model provides the highest classification performance (96.1% accuracy for normal vs. COVID-19, 99.5% accuracy for COVID-19 vs. viral pneumonia, and 99.7% accuracy for COVID-19 vs. bacterial pneumonia).

In the field of pediatric pulmonary imaging, an interesting study by Liang and Zheng (2020) proposed a deep-learning network that combines residual structures and dilated convolution with the purpose of diagnosing pneumonia by using raw chest X-ray images from children from 1 to 5 years of age. Their data set involved a total of 6,090 chest X-ray images, 4,117 images from children with pneumonia, and 1,973 images from healthy infants. Their method obtained an accuracy of 90%, a recall rate of 96.7%, and the F1-score of 92.7% on pneumonia classification tasks. A similar approach, developed by Saraiva. et al. (2019), obtained an average accuracy of over 95% on a binary classification problem for detecting pneumonia cases from chest X-ray images. Our results are competitive when compared to those obtained by using both X-ray and LUS imaging modalities, both in pediatric studies regarding cases of pneumonia (Liang and Zheng, 2020) and in recent investigations involving patients with different kinds of pneumonia (i.e., COVID-19 and bacterial pneumonia). In fact, our Inception-ResNet-v2 model achieves 97.75% accuracy, 97.75% sensitivity, and 97% specificity for healthy vs. bronchiolitis, whereas the Inception-v3 model provides the best results with 91.5% accuracy, 91.5% sensitivity, and 95.86% specificity for healthy vs. bronchiolitis vs. bacterial pneumonia.

Interestingly, a study from Correa et al. (2018) examined brightness profiles of pleural lines in children younger than 5 years of age; they were associated with three possible diagnoses: pneumonia, healthy, and bone. The authors used a feed-forward neural network composed of three layers and sigmoid as an activation function. Their approach achieves a sensitivity of 90.9% and a specificity of 100% in detecting vectors associated with pneumonia consolidation. The results of their study support our findings that filters of the first convolutional layers learned by the optimal model respond mainly to color features (see section 3.3.1), with brightness being expressible as a linear combination of RGB color components. Furthermore, when inspecting the convolutional filters (Figure 3), and activation maps (Figure 4) of the first convolutional layers, we can see that they also responded to edges and texture-like patterns (see sections 3.3.1 and 3.3.2). This observation, when considered together with the fact that the Grad-CAM highlights areas of medical interest, suggests that, when taking its decision over the classification outcome, the network looks predominantly at those specific patterns.

A fair amount of research is devoted to the use of deep-learning approaches for analysing LUS images by focusing on training deep neural networks on isolated, hand-crafted features that are considered diagnostically valid, i.e., A-lines, vertical artifacts, pleural lines (Carrer et al., 2020), pleural effusions, and also consolidations, with vertical artifact (i.e., B-lines) detection the most common task (Kulhare et al., 2018; Wang et al., 2019; van Sloun and Demi, 2020). See McDermott et al. (2021), for a review. Conversely, we opted for applying deep neural networks to raw images, because this usually permits avoiding the introduction of typical errors caused by inaccurate results of image pre-processing steps (e.g., image segmentation and decomposition) and cognitive biases or confidence in spatial relationships between pixels and could lead to the discovery of unexpected associations that would remain otherwise undetected (Poplin et al., 2018). Furthermore, feeding the deep neural network with raw images enables us to take better advantage of deep-learning potential: (1) the automatic detection of the appropriate predictive visual features from the training data enables the feature extraction without requiring features to be hand-engineered; (2) feature interaction and hierarchy can be exploited jointly within the intrinsic deep architecture of a neural network; (3) the three steps of feature selection, feature extraction and supervised classification, can be realized within the optimization of the same deep architecture, and the performance can be tuned more easily in a systematic fashion. As a result, the models achieved very high performances in both classification tasks. We can also observe that the optimal model appears to be able to distinguish the specific appearance of visual features (such as B-lines, consolidations etc.) in different diagnoses, rather than merely being able to look for the presence or absence of them. In fact, growing evidence indicates that artifacts can have a different semeiotic, according to each disease (Soldati et al., 2016, 2019).

### 4.4. Limitations

**Figure 8:**
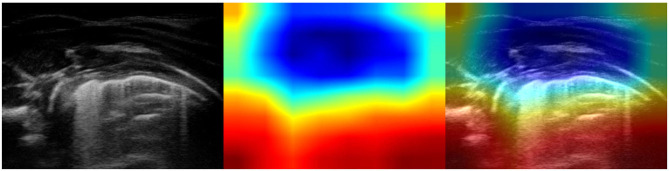
Example of lung ultrasound image of a healthy lung incorrectly classified as bronchiolitis by the best performance model (Inception-v3). **(Left)** Original LUS image; **(Middle)** Class activation mapping produced by the Grad-CAM visualization; **(Right)** The class activation mapping overlaid transparently on the original LUS image. Note that red and orange regions correspond to high scores for the predicted class and highlight multiple, confluent long vertical artifacts. These visual features are typical of bronchiolitis but can also be observed in very young healthy infants (~ 6 months of life). Therefore, the optimal model “mistake” results in the recognition of multiple long vertical artifacts.

Although our data set contains a large number of images, it suffers from a limitation due to the relatively small number of individuals, particularly children with pneumonia. It is important to observe that only two out of three groups of children were matched by age: healthy infants (age: 2.83 ± 2.89 months) and infants with bronchiolitis (age: 2.50 ± 2.67 months). The age of children suffering from bacterial pneumonia (age: 6.17 ± 6.77 years) is statistically different from that of the subjects in the other two groups. The patient populations of our study reflect real-life clinical situations, i.e., bronchiolitis tends to affect younger infants with respect to bacterial pneumonia. Although the obtained results are promising, they might not be generalizable to patients of all ages. The main features of lung pathology (e.g., consolidations, pleural effusions, and bronchograms) were demonstrated to be the same across all age groups, from newborns to adults, the only exception being vertical artifacts. Although vertical artifacts are traditionally considered as a sign of interstitial disease, rather than one of a healthy lung, they can be morphologically different in a healthy lung, in a pathological lung and in the case of diverse diseases (Soldati et al., 2016, 2019). For instance, we recently showed that the healthy younger infants (~6 months of life) can have a lung ultrasound pattern characterized by multiple vertical lung artifacts (Buonsenso et al., 2020d). This is probably due to the immature development of the lung in the first months of life. In line with these results, our models, misinterpreted some cases of healthy infants, whose images were characterized by the presence of multiple, often confluent, vertical artifacts (see Figure 8, for an example), such as with bronchiolitis. In a real-life scenario the “age-effect” should have no impact on the interpretation of LUS features. However, from a methodological point of view, further studies should address this issue and, in order to achieve better classification performance, should therefore focus on training deep neural networks, by using more diverse examples of LUS images that contain vertical artifacts from both healthy infants and patients with interstitial disease. In fact, the correct diagnosis of bronchiolitis and, in particular, the possibility of making a distinction between acute bronchiolitis and pneumonia are crucial in young infants because this age group bears the highest global mortality rate for both bronchiolitis and pneumonia. As these two conditions require different management (antibiotics are needed only in case of pneumonia), the differentiation of these two conditions by point-of-care lung ultrasound, particularly in poor settings, could be particularly relevant from a global health perspective.

**Figure 9:**
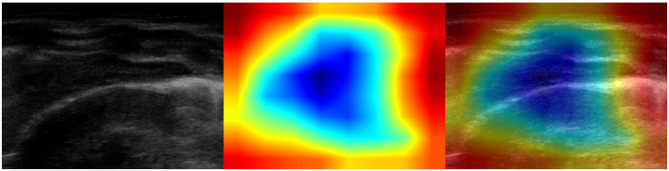
Example of lung ultrasound image of a healthy lung incorrectly classified as bacterial pneumonia by the best performance model (Inception-v3). **(Left)** Original LUS image; **(Middle)** Class activation mapping produced by the Grad-CAM visualization; **(Right)** The class activation mapping overlaid transparently on the original LUS image. Note that red and orange regions corresponds to high scores for the predicted class and highlight what might be confused with consolidations, being the image really dark. This mistake helps us to better understand how the training set should be balanced with a more comprehensive inclusion of the different visual features.

Another limitation of our study is certainly related to data acquisition. We did not set up a standard procedure for obtaining images from the ultrasound probe, i.e., zoom, gain, mechanical index, and focus positioning were perhaps not always the same in every patient. When those images were either oversaturated or extremely dark, the model made a few objective mistakes by misinterpreting some images of healthy lungs as images that displayed lungs with bacterial pneumonia (Figure 9): input perturbations might have been confused with consolidations by the model. The ability to set the proper settings depends on the experience of the sonographer, and different settings can lead to different lung ultrasound patterns, even when scanning the very same areas. Establishing standard settings is probably one of the main challenges of using lung ultrasound imaging. Only recently, and much later with respect to the time of our data acquisition, researchers of the Italian Academy of Thoracic Ultrasound proposed a standardization with respect to the use of LUS in the management of COVID-19 patients by specifying imaging and device settings, among the other procedures, with the aim of reaching a more globally unified approach for comparisons between different human- and computer-aided studies; hence a better understanding of the role of LUS in the diagnosis of COVID-19 (Soldati et al., 2020).

### 4.5. Applications and Future Directions

At present, we have implemented a simple decision support tool (computer aided diagnosis system or CADx) that can assist medical doctors in formulating their diagnoses: given as input a sequence of LUS images, our system is able to suggest a diagnosis on the basis of the majority of the CNN classification votes obtained over all images belonging to a patient. The diagnosis spans over the three above-mentioned diagnostic groups: healthy subjects and patients with bacterial pneumonia or bronchiolitis. The final decision of the diagnosis is eventually taken by the medical personnel, with the help of the Grad-CAM that is displayed for each LUS image belonging to a specific patient. The explainable method gives quantitative feedback (in terms of probability) on the rationale behind the decision taken by the model hence might also be useful for instructional purposes (Muse and Topol, 2020).

When validated adequately, the method we propose could help to simplify and accelerate the diagnosis of pulmonary diseases, and if extended, in the future, might enable the differentiation among different types of bacterial and viral pneumonia (including COVID-19). Considering the fact that deep learning usually performs better when the available data points represent well the distribution at end, our future works will be to collect ultrasound image sets that are greater and better balanced, with respect to age groups, ethnicity and device settings, despite the fact that methodical data collection is time-consuming and challenging due to the paucity of data. Particular attention could be given to the anomaly detection problem (Chandola et al., 2009). In most classification tasks the presence of one or more negative (alien or abnormal) classes constitutes a challenge, especially considering the lack of data of these classes and the openness of the problem. A typical solution to overcome this problem is to use supervised anomaly detection thus build a predictive model for normal vs. anomaly classes that might then contain data belonging to other illnesses not contemplated by the actual classification. Different techniques have been used to address the anomaly detection problem and could be adopted in future works: generative adversarial networks for anomaly detection (Schlegl et al., 2019) and the methods based on one class classification (Ruff et al., 2018). Many other approaches could be investigated to refine our results: careful choices for the topology of a neural network, extensive use of transfer learning in compatible domains, exploring other deep-learning methods, for example, with the objective of emphasizing robustness and explainability (Roberts and Tsiligkaridis, 2020). Additionally, our approach might be combined with the classification of disease severity (Supino et al., 2019) or with LUS image quality assessment module (Baum et al., 2021).

## 5. Conclusions

In conclusion, our study represents a first step for the development of a CADx system that is able to assess and classify pediatric LUS images as belonging to different pulmonary diseases. When extensively validated, such a system could reduce the daily burden of clinicians, could assist them in making more accurate diagnoses, and could enable better comparisons of images obtained during follow-up. Moreover, CADx systems could provide a second opinion to expert radiologists and remote training assistance, which could be particularly useful in remote geographic areas with a limited availability of diagnostic tools.

## Data Availability Statement

The raw data supporting the conclusions of this article will be made available by the authors, without undue reservation.

## Ethics Statement

The studies involving human participants were reviewed and approved by ethics committee of the Fondazione Policlinico Universitario A. Gemelli IRCCS Rome, Italy. (prot 36173/19, ID 2729). Written informed consent to participate in this study was provided by the participants' legal guardian/next of kin.

## Author Contributions

SM, PV, and DB conceptualized the study. DB, CD, and RM performed the lung ultrasound scans. SM was responsible for the deep-learning studies. SM and DB wrote the first draft of the manuscript. All authors read and approved the last version of the manuscript.

## Conflict of Interest

The authors declare that the research was conducted in the absence of any commercial or financial relationships that could be construed as a potential conflict of interest.

## Publisher's Note

All claims expressed in this article are solely those of the authors and do not necessarily represent those of their affiliated organizations, or those of the publisher, the editors and the reviewers. Any product that may be evaluated in this article, or claim that may be made by its manufacturer, is not guaranteed or endorsed by the publisher.
